# TDR: (Back) Making a Difference

**DOI:** 10.1371/journal.pntd.0002531

**Published:** 2014-01-09

**Authors:** John C. Reeder

**Affiliations:** Director of the Special Programme for Research and Training in Tropical Diseases (TDR), The World Health Organization, Geneva, Switzerland; Swiss Tropical Institute, Switzerland

The Special Programme for Research and Training in Tropical Diseases, or “TDR” as it is popularly known, has been a prominent player in the global health field for almost 40 years. Thousands of scientists worldwide have benefited from training and research support facilitated by TDR and the organization has an outstanding list of accomplishments in enabling research evidence to be translated into more effective disease control impact. However, TDR's many passionate supporters have been worried over the last few years to hear of the apparent decline of the organization and even rumours of its demise. But while there have certainly been challenges of late, as Mark Twain famously said, reports of our death have been greatly exaggerated! Today TDR stands strong again, refocused on its mission, reorganized into a more efficient and cost-effective structure and now embarking on a new and innovative programme of activities that will see it back once more on the frontline of the fight against infectious diseases of poverty.

TDR was formed in 1974 with the two interdependent objectives of developing improved tools for the control of tropical diseases and strengthening the research capability of affected countries themselves. Its purpose was to take on the world's most neglected diseases, affecting millions of people in poor countries, and TDR contributed to the development of critically needed new diagnostics, drugs, and drug combination therapies for these diseases [1]. An innovative compound screening network identified the potential of ivermectin for river blindness (onchocerciasis). TDR's early support for Chinese research into artemisinin derivatives for malaria helped introduce an ancient remedy onto global research agendas. Field and socio-economic research guided treatment of socially stigmatized diseases such as leprosy and identified simple tools, such as bed nets, to prevent malaria. Empowering community members to manage simple health and disease-control measures was found to be so effective, it is now being used among 60 million Africans to manage river blindness. TDR also helped incubate and launch several new public–private partnerships for drug and diagnostics development and led an initiative in global drug discovery research networks. During all of this, building research capacity in low- and middle-income countries was at the core of the programme. TDR supported long-term development of research institutions in disease-endemic countries and trained thousands of scientists. In recognition of this sustained and significant contribution to health, TDR was the proud recipient of the 2011 Gates Award for Global Health. This was, however, a bittersweet moment, as by this time the global financial crisis was hitting hard and decisions taken by TDR in more promising economic conditions came back to haunt it.

In many ways TDR was a victim of its own success. The demand for new treatments and diagnostics for diseases affecting neglected populations was at an unprecedented high, and the track record of TDR attracted many demands for engagement. This led to a very ambitious expansion of activities, particularly in research and development projects and sponsorship of large clinical trials, and this in turn necessitated a rapid expansion of staffing at headquarters. All this was of course predicated upon income continuing to follow its upward trend, and when it did not, calamity struck. In the wake of a very large financial shortfall, staffing had to be dramatically reduced, and spending curtailed. Priority had to be given to clinical studies with ethical implications for recruited patients and for trying to draw these trials to a satisfactory close. Unfortunately, this resulted in support being withdrawn prematurely from some other worthy projects, and while many investigators were resilient enough to carry on by other means, regretfully, some projects had to be abandoned, undoubtedly leaving a bruise on the reputation of TDR. It is noteworthy though that, even during this difficult period, 207 papers published in 2012 acknowledged TDR support, 65% of these with a first author from a disease endemic country.

Despite the obvious pain for partners and staff alike, many hard lessons were learned from this game-changing situation. Fundamental questions were asked, including whether there was still a need for TDR in the present environment. The resounding response from the community to this question was yes, TDR still had much to offer [2], but only if it took careful stock of the current global health research landscape and the vast changes that had been occurring, then looked realistically at its own strengths and weaknesses, to focus its presence where it could have the most impact upon the burden of disease in the most neglected populations. This was the basis of the new TDR strategy [3], a slim but carefully thought-out document articulating the basic principles on which the organization will build its activities. At the front of this strategic refocus is a clear mission statement: “To foster an effective global research effort on infectious diseases of poverty and promote the translation of innovation to health impact in disease endemic countries.” To achieve this goal, TDR is returning to its proven key strengths of increasing the capacity of individuals and institutions to perform research related to their priority health issues, engaging disease endemic countries in setting the health research agenda, and fostering research and developing innovative knowledge that leads to health improvement. The focus will remain with infectious diseases of poverty and priority is being given to areas where the most direct impact can be made on health. To do this, we are moving away from research and development for drugs and diagnostics, as many extremely capable organizations, including a number of which TDR helped establish, are now working in this space. We are now concentrating on research for the effective implementation of existing and new interventions in low-and middle-income countries, bringing research and innovation into the service of those burdened most heavily by neglected infectious diseases.

Such forward-looking plans could not be considered, however, if the organizational foundation had not also moved forward. Using the needs of the new strategy as a blueprint, TDR has now reorganized into a leaner structure. The greatly reduced cost of such a structure means a higher percentage of income will be made available for training and research in the field, giving excellent value for their money to our donors and more opportunities for scientists from disease endemic countries. Questions have been raised about whether such a reduced structure can still maintain the critical mass to have real impact, but it will not have escaped the attention of older observers that TDR made a very significant impact in the 1980s and 90 s with just such a team [4]. The key to increasing impact while reducing organizational size is in the method of engagement. Working through partnership is absolutely essential for success, and TDR will be concentrating on its role as a facilitator and enabler of research, rather than acting as the primary investigator.

Now that the lessons of the recent past have been well learned and we have a strategically focused mission and an efficient, fit-for-purpose organizational structure, what can the research community expect from TDR? During 2013, the focus was still on the completion of a number of large trials, to fulfil our obligation to these important legacy projects, though new schemes also emerged as a foretaste of future plans. The IMPACT fellowships that help researchers gain new skills in order to engage directly with disease control questions have already been launched [5], and the ever-popular small grant scheme through WHO regional offices has been renewed. Calls have now started to emerge for an exciting new programme of activities to be supported in the 2014/15 biennium.

Research capacity-strengthening opportunities are once more available to increase the critical mass of researchers in low- and middle-income countries through formal research training (master's and PhD), but there is a focus on these training projects developing the skills and evidence needed to directly address local health problems. Other output-oriented training is targeted directly at those working in public health interventions, to increase the generation and uptake of evidence to strengthen implementation. Support is also being given to regional training centres to promote South–South networking and engagement.

Intervention and implementation research is being targeted into areas that support improved disease control in endemic countries. All research will be closely entwined with capacity development, and there will be no research without capacity development and no capacity development without research leading to health impact. Reducing the vulnerability of disease control programmes to drug and insecticide resistance through improved surveillance markers, improving control of dengue and Chagas disease through innovative ecosystem management, improving strategies for community-based management of childhood febrile illness, and evaluating the use of social entrepreneurship for sustainable disease control and prevention are just a few of the research areas to be explored. TDR will also use its neutral position as a United Nations platform organization to work with the research community to identify research priorities, help close gaps in intervention evidence through harmonization of methodologies, consolidate evidence for policy recommendations, and improve accessibility to drug safety data.

This is a time of great potential for the control of infectious diseases of poverty. Political interest is high, new tools are available, and many capable and committed organisations are now working together in a once barren landscape. TDR has stood back up, dusted itself off, and brought its unique position and long experience back to this fight. We look forward to working with you to make a difference.
